# Complications of Unilateral Biportal Endoscopic Spinal Surgery for Lumbar Spinal Stenosis: A Systematic Review of the Literature and Meta‐analysis of Single‐arm Studies

**DOI:** 10.1111/os.13437

**Published:** 2022-11-17

**Authors:** Bin Wang, Peng He, Xiowei Liu, Zhengfang Wu, Bin Xu

**Affiliations:** ^1^ Department of Orthopedics Jingling Hospital, Medicine College, Nanjing University Nanjing China

**Keywords:** Complications, Lumbar spinal stenosis, Single‐arm Meta‐analysis, System review, Unilateral biportal endoscopic

## Abstract

**Purpose:**

This article aims to summarize the incidence of these complications through a meta‐analysis, analyze the causes of complications and provide clinical promotion and recommendations.

**Methods:**

Databases and retrieval platform including PubMed, Web of science, Springer link, Cochrane clinical trials, ProQuest, ScienceDirect, Europe PMC, Wiley online, OVID, Clinical trials, CNKI and WanFang, and supplement the literature through Google Scholar, collect all the unilateral biportal endoscopy (UBE) controlled trials and non‐controlled trials of UBE in the treatment of lumbar spinal stenosis (LSS). The search time limit is from January 1, 2000 to December 25, 2021. After two reviewers independently screened the literature, extracted data and evaluated the risk of bias in the included studies, meta‐analysis was performed using Stata 15.1 software.

**Results:**

Finally, 24 studies were included, including 999 patients. The results of a single‐arm rate meta‐analysis showed that the overall complication rate of UBE treatment of LSS was 6.27% [95% CI (0.0412, 0.0876)], and the incidence of dural tear was 2.49% [95% CI (0.0133, 0.0390)], the incidence of transient paresthesia was 0.14% [95% CI (0.0000, 0.0072)], the incidence of postoperative spinal epidural hematoma was 0.27% [95% CI (0.0000, 0.0096)], the incidence of postop headache, inadequate decompression, root injury and infection was 0.00%.

**Conclusion:**

Current evidence shows that the complication rate of UBE in the treatment of LSS is low, mainly due to dural tears. Limited by the number and quality of included studies, the above conclusions still need to be confirmed by more studies.

## Graphical abstract

The graphical abstract of Single‐arm meta analysis of UBE for LSS.

- Analysis of the complication rate of unilateral biportal endoscopic in the treatment of lumbar spinal stenosis.
- Analyze the causes of complications.
- Advice on reducing complications.

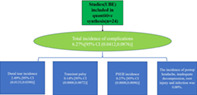

## Introduction

Lumbar spinal stenosis (LSS) is a common degenerative disease of the lumbar spine, accounting for 8% to 11% of all degenerative diseases requiring surgical treatment.^1^ The disease can be caused by any type of spinal canal, nerve root canal or intervertebral foramen, the symptoms are lower back or lower extremity pain caused by nerve root compression. Although most patients choose conservative treatment in the early stages, as the disease progresses, due to the serious impact on the quality of life, most patients have to choose surgical treatment to relieve symptoms.

Clinically, the treatment of LSS has undergone the transformation and development from open to minimally invasive, mainly because open surgery may cause greater damage to paravertebral anatomical structures such as muscles and facet joints, which may lead to postoperative complications. The biomechanical stability of the spine is reduced, and long‐term complications such as degeneration of adjacent vertebral bodies occur, and minimally invasive spine surgery emerges as the times require.^2^, ^3^ At present, the most popular minimally invasive surgery is mainly the foraminal technique. However, the intraoperative operation space of this technique is small, the decompression may be incomplete, and the learning curve is steep, which is not friendly to young clinicians.^4^ Therefore, unilateral biportal endoscopy (UBE), which was proposed by Kambin and Sampson in 1986, has re‐entered people's field of vision in recent years under the improvement of Korean scholars.^5^, ^6^

The reason why UBE is currently recognized and popular is mainly because: (i) it has the characteristics of flexible operation in open surgery and a clear magnified field of view in minimally invasive surgery; (ii) it can be used similar to conventional foraminal scope; (iii) because of the multifidus interstitial approach, the damage to the paraspinal muscles is less; (iv) the number of intraoperative fluoroscopy is less, which reduces the radiation level to the patient; and (v) surgery has a wide range of indications and can be used to treat degenerative diseases such as lumbar disc herniation, I ~ II° lumbar spondylolisthesis. With the popularization of UBE technology, some problems have also arisen, mainly in the complications of surgery. After our literature search and screening, we found that these complications are dural tear, transient paresthesia, postoperative spinal epidural hematoma (PSEH), postop headaches, inadequate decompression, root injury and infection. The purpose of this study is as follows: (i) to review the current clinical studies on the use of UBE for the treatment of LSS, and to provide experience for clinicians who perform this procedure; (ii) to summarize the complications of LSS using UBE technology and analyze the incidence of these complications by meta‐analysis; and (iii) to analyze the possible causes of intraoperative complications in UBE.

## Methods and Materials

### Search Strategy

To perform comprehensive retrieval strategy, we systematically searched relevant studies published in electronic databases including PubMed, Web of science, Springer link, Cochrane clinical trials, ProQuest, ScienceDirect, Europe PMC, Wiley online, OVID, Clinical trials CNKI and WanFang, and supplement the literature through Google Scholar, collect all the unilateral dual channels RCT, non‐RCT and single‐arm trials of endoscopic technique in the treatment of LSS. The search time limit is from January 1, 2000 to December 25, 2021. The search is carried out by combining subject terms and free words. The search strategy is determined after multiple pre‐searches, and the search terms are adjusted according to the specific database. Search terms include: “biportal endoscopic spinal surgery” OR “two portal endoscopic spinal surgery” AND “Lumbar spinal stenosis”.

### Inclusion and Exclusion Criteria

The criteria for inclusion article: (i) research type: RCT, non‐RCT or single‐arm clinical research on the treatment of LSS with UBE; (ii) research objects: patients who have clear clinical symptoms and are diagnosed with LSS such by MRI or X‐ray; and (iii) intervention measures: All patients with LSS were treated with UBE surgery.

Exclusion criteria included: (i) non‐English literature; (ii) multiple reports from a single center, studies with the largest sample size without repetition after reading the full text; (iii) for multiple similar studies in the same unit, exclude duplicate cases after reading the full text; and (iv) the patients have serious cardiovascular and cerebrovascular diseases, mental diseases, malignant tumors, etc.

### Data Extraction

Two investigators independently screened the literature according to the inclusion and exclusion criteria, extracted data and cross‐checked them. In cases of disagreement, they were discussed and resolved, and the third investigator's opinion was sought if necessary. If the information in the research report is incomplete, the author was contacted to obtain further information; a pre‐designed data extraction table was used to extract the information. The main information extracted from the data includes: (i) general information of the included study, including title, author, year of publication, etc.; (ii) research characteristics, including study area, sample size, age, operation time, follow‐up time, etc.; (iii) concerned outcome indicators and outcome measurement data; and (4) relevant data for bias risk evaluation.

### Quality Assessment

In this study, the ethodological index for non‐randomized studies (MINORS)^7^ was used to evaluate the risk of bias. Since this study only evaluated the single‐arm rate outcome, only the first eight items MINORS were used for evaluation.

### Outcome Indictor

In the present meta‐analysis, outcome indicators are included: (i) postop headache: Anesthesia, insufficient body fluid supplement and other reasons cause postoperative headaches in patients; (ii) dural tear: intraoperative or postoperative cerebrospinal fluid leakage; (iii) transient paresthesiay: transient limb numbness after surgery; (iv) PSEH: Postoperative radiographic data showed epidural hematoma; (v) inadequate decompression: postoperative symptoms did not improve significantly; (vi) root injury: injury to nerve roots during surgery, resulting in corresponding sensory and motor dysfunction; and (viii) infection: postoperatively, the incision of the patient did not heal well, there was an inflammatory reaction, and the bacterial test of the drainage tube was positive.

### Statistical Analysis

The statistical software Stata15.1 (StataCorp, College Station, TX, USA) was used to analyze the complications. The I^2^ test was used to test the heterogeneity of the included literature. According to the Cochrane manual, I^2^ > 50% was considered heterogeneity, and a random effects model was selected for combined statistical analysis; when I^2^ < 50%, heterogeneity did not exist. A fixed‐effects model was chosen for data processing and analysis. Since the incidence of complications in this meta‐analysis is relatively small (0 < *P* < 0.2), metaprop is used to perform double arcsine transformation of the data and then statistically merge.^8^, ^9^

### Sensitivity Analysis

Sensitivity analysis was performed on the included studies using the metain command in tata15.1 software.

### Publication Bias

The metafunnel command in the Stata15.1 software was used to draw the funnel diagram, and judge the publication bias subjectively by observing the symmetry of the funnel diagram. If the funnel diagram is symmetrical, there is no publication bias, and the Egger's test is used to objectively judge whether there is publication bias, *P* > 0.05 Explain funnel chart symmetry.

## Results

### Selection of Studies

**Fig. 1:**
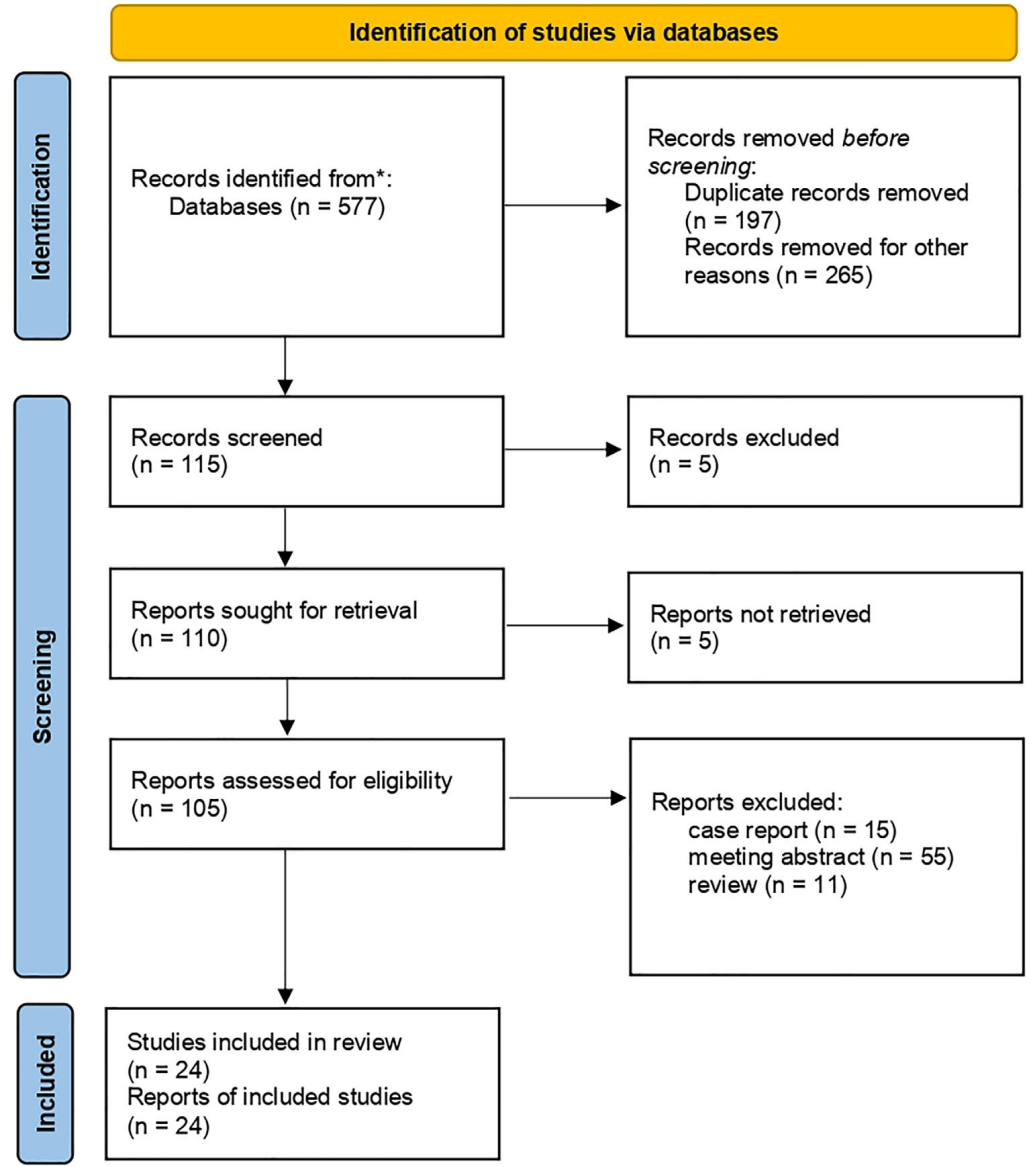
Flow diagram for the meta‐analysis method. * database or platform and articles number: PubMed(n = 103); Web of science: (n = 100); Cochrane clinical trials: (n = 9); ProQuest:(n = 29); ScienceDirect: (n = 1); Europe PMC: (n = 144); Wiley online:(n = 2); Clinical trials: (n = 1); CNKI: (n = 28); WanFang: (n = 16); Google scholar: (n = 144)

A flow chart for the inclusion of studies is shown in Fig. 1. A total of 577 related documents were obtained in the preliminary inspection, including PubMed: 103, Web of science: 100, Cochrane clinical trials: nine, ProQuest: 29, ScienceDirect: one, Europe PMC: 144, Wiley online: two, Clinical trials: one, CNKI:28, WanFang: 16, Google scholar: 144. After excluding duplicate documents and reading the title and abstract, 462 articles were obtained; 115 articles remained; further reading the full text for re‐screening, the remaining 81 articles were excluded, 24 studies were included eventually.

### Characteristics of the Studies Included in the Meta‐analysis

The basic characteristics of the included studies are shown in Table 1. A total of 24 studies were included, including 10 single‐arm studies and14 controlled trials which include two RCT studies, two Prospective studies and 10 retrospective studies. Ten of them were Korean studies, one Thai study, one Hungarian study, eight Chinese studies, one Ukrainian study, one Japanese study and one Saudi Arabian study. A total of 999 patients with lumbar spinal stenosis were included. All patients have similar basic diseases, treatment methods and other basic characteristics.

**TABLE 1:** Basic characteristics of included studies

Author	Country/Region	Year	Design	Sample size (M/F)	Age (years)	Operation time (min)	Pressure (mmHg)	Blood loss (mL) /ΔHb (g/L)	Complications
Eum *et al*.^12^	Korea	2016	Retrospective	58 (18/40)	63.4 ± 7.4	68.9 ± 16.1	20–30		A (3); B (2); C (2); D (1)
Torudom *et al*.^33^	Thailand	2016	Retrospective	30 (11/19)	56 ± 6.2	98.3 ± 14.3			C (2); E (1)
Kim *et al*.^27^	Korea	2018	Retrospective	55 (26/29)	70.7 ± 14.3	53 ± 11.5	30		B (2); D (1)
Heo *et al*.^34^	Korea	2018	Prospective	46 (18/28)	65.8 ± 8.9	61.1 ± 5.2			B (1); D (1)
Park *et al*.^10^	Korea	2019	Retrospective	60 (31/29)	67.6 (41–91)	83.8 ± 37.9	40		B (3); D (1); E (2)
Choi *et al*.^14^	Korea	2019	Retrospective	35 (14/21)	65.4 ± 11.8		25–30	(−2.5 ± 0.7) g/L	
Heo *et al*.^35^	Korea	2019	Retrospective	37 (15/22)	66.7 ± 9.4	62.4 ± 5.7			B (1); D (1)
Kang *et al*.^36^	Korea	2019	RCT	32 (18/14)	65.1 ± 8.6	36 ± 11	30		D (1)
Zhang *et al*.^37^	China	2019	Retrospective	5 (3/2)	68.5	110.6 ± 18.9	30	15.2 ± 9.7 mL	
Pao *et al*.^11^	China (Taiwan)	2020	Retrospective	81 (38/43)	70.2 ± 10.8	89 ± 56.9			B (4); C (1); D (1); E (1)
Czigleczki *et al*.^13^	Hungary	2020	Retrospective	21 (9/12)	66.5 (30–83)		20–30		A (3); B (2); E (1)
Min *et al*.^38^	Korea	2020	Retrospective	54 (27/27)	65.74 ± 10.52	53.68 ± 6.75			B (2); D (1)
Park *et al*.^39^	Korea	2020	RCT	32 (13/19)	66.2 (41–80)	67.2 ± 19.8	30–40		B (2); D (1)
Kim *et al*.^40^	Korea	2020	Retrospective	30 (13/17)	64.23 ± 5.26	58.10 ± 6.04		53.63 ± 10.08 mL	B (1)
Fishchenko *et al*.^41^	Ukraine	2020	Retrospective	67 (37/30)	45.80 ± 14.18	71.3 ± 21.9		34.8 ± 16.2 mL	B (4)
Ito *et al*.^42^	Japan	2021	Retrospective	42 (28/14)	66.3 ± 12.3	57 ± 10.3	30		B (2)
Aygun *et al*.^43^	Turkey	2021	Prospective	77 (44/33)	64.64 ± 10.09	57.74		49.47 mL	
Chen *et al*.^44^	China	2021	Retrospective	13 (7/6)	63.5 (48–81)	75.1 (55–100)		48.8 (25–80) mL	B (1)
Tuo *et al*.^45^	China	2021	Retrospective	22 (12/10)	59.1 ± 11.7			48.2 ± 7.2 mL	B (3); D (1)
Wang *et al*.^46^	China	2021	Retrospective	Central: 28 (15/13)	Central: 67.79 ± 6.29	Central: 120.75 ± 9.79		Central: −21.54 ± 9.79 g/L	C (5)
				Lateral: 36 (17/19)	Lateral: 62.75 ± 8.02	Lateral: 106.4 ± 12.99		Lateral: −21.00 ± 7.67 g/L	
Zhao *et al*.^47^	China	2021	Retrospective	34 (16/18)	65.71 ± 10.55	65.30 ± 9.58		<50 mL	
Kang *et al*.^48^	China	2021	Retrospective	50 (22/28)	64.97 ± 9.83	62.59 ± 7.10		47.15 ± 9.20 mL	B (2); F (3); G (2)
Wang *et al*.^49^	China	2021	Retrospective	23 (13/10)	61.52 ± 4.09	66.52 ± 3.51		46.96 ± 4.19 mL	B (1); C (1)
Gu *et al*.^50^	China	2021	Retrospective	31 (11/20)	62.8 ± 8.1	55.1 ± 11.7			B (1)

Abbreviations: A, postop headache; B, dural tear; C, transient paresthesia; D, PSEH; E, inadequate decompression; F, root injury; G, infection; RCT, Randomized controlled study.

### Methodological Study Quality Assessment

See Table 2 for the evaluation results of the risk of bias of the included studies.

**TABLE 2:** Results of risk assessment of bias in included studies

Study	A clearly state aim	Inclusion of consecutive patients	Prospective collection of data	Endpoints appropriate to the aim of the study	Unbiased assessment of the study endpoint	Follow‐up period appropriate to the aim of the study	Loss to follow up less than 5%	Prospective calculation of the study size
Eum *et al*.^12^	2	2	2	2	1	2	0	0
Torudom *et al*.^33^	2	2	2	2	1	2	2	0
Kim *et al*.^27^	2	2	2	2	1	2	0	0
Heo *et al*.^34^	2	2	2	2	1	2	0	0
Park *et al*.^10^	2	2	2	2	2	2	2	2
Choi *et al*.^14^	2	2	2	2	1	2	0	0
Heo *et al*.^35^	2	2	2	2	1	2	1	0
Kang *et al*.^36^	2	2	2	2	1	2	0	0
Zhang *et al*.^37^	2	2	2	2	1	2	2	0
Pao *et al*.^11^	2	2	2	2	1	2	2	0
Czigleczki *et al*.^13^	2	2	2	2	1	2	2	0
Min *et al*.^38^	2	2	2	2	1	2	2	0
Park *et al*.^39^	2	2	2	2	2	2	0	2
Kim *et al*.^40^	2	2	2	2	1	2	2	0
Fishchenko *et al*.^41^	2	2	2	2	1	2	2	0
Ito *et al*.^42^	2	2	2	2	1	2	2	0
Aygun *et al*.^43^	2	2	2	2	1	2	2	0
Chen *et al*.^44^	2	2	2	2	1	2	2	0
Tuo *et al*.^45^	2	2	2	2	1	2	2	0
Wang *et al*.^46^	2	2	2	2	1	2	2	0
Zhao *et al*.^47^	2	2	2	2	1	2	2	0
Kang *et al*.^48^	2	2	2	2	1	2	2	0
Wang *et al*.^49^	2	2	2	2	1	2	2	0
Gu *et al*.^50^	2	2	2	2	1	2	2	0

*Note*: The items are scored 0 (not reported), 1 (reported but inadequate) or 2 (reported and adequate).

### Meta‐Analysis Results

#### Primary Outcomes

##### Overall Complication Rate

**Fig. 2:**
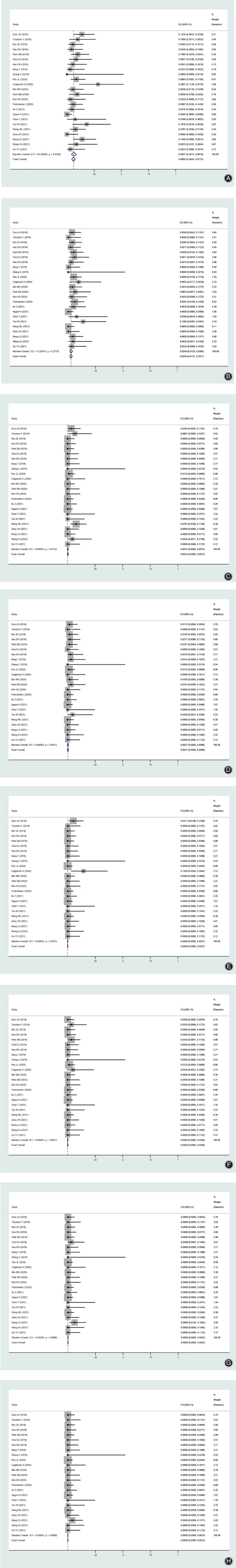
(A) Forest plot of overall complication rate. (B) Forest plot of dural tear rate. (C) Forest plot of transient paresthesia rate. (D) Forest plot of PSEH rate. (E) Forest plot of postop headache rate. (F) Forest plot of inadequate decompression rate. (G) Forest plot of root injury rate. (H) Forest plot of infection rate

Except for four studies that did not report surgical complications,^15^, ^18^, ^26^, ^30^ all other studies reported the occurrence of surgical complications, and the heterogeneity of different studies was statistically different (I^2^ = 44.55%, *P* = 0.01), but because the single‐arm meta itself has large heterogeneity, and sensitivity analysis and publication bias showed that no literature had a large interference with the results, so subgroup analysis was not performed, and a random‐effects model was used for meta‐analysis. The overall complication rate of UBE treatment of LSS was 6.27% [95% CI (0.0412, 0.0876)] (Fig. 2A).

##### Dural Tear Rate

A total of 17 studies reported the occurrence of dural tear during surgery,^10^, ^12^, ^13^, ^14^, ^16^, ^19^, ^20^, ^21^, ^22^, ^23^, ^24^, ^25^, ^27^, ^28^, ^31^, ^32^, ^33^ and there was no heterogeneity among the studies (I^2^ = 13.23%, *p* = 0.28). A random‐effects model was used for meta‐analysis. The incidence of dural tear was 2.49% [95% CI (0.0133, 0.0390)] (Fig. 2B).

##### Transient Paresthesia Rate

A total of five studies reported transient paresthesia during surgery,^10^, ^11^, ^19^, ^29^, ^32^ and there was no heterogeneity among studies (I^2^ = 0.00%, *P* = 0.57). A random effects model was used for meta‐analysis. The incidence of transient paresthesia was 0.14% [95% CI (0.0000, 0.0072)] (Fig. 2C).

##### PSEH Rate

A total of ten studies reported the occurrence of PSEH during surgery,^10^, ^12^, ^13^, ^14^, ^16^, ^17^, ^19^, ^21^, ^22^, ^28^ and there was no heterogeneity between studies (I^2^ = 0.00%, *P* = 0.98). A random effects model was used for meta‐analysis. The incidence of PSEH was 0.27% [95% CI (0.0000, 0.0096)] (Fig. 2D).

#### Secondary Outcomes

##### Postop Headache Rate

A total of two studies reported postop headache during surgery,^10^, ^20^ and there was no heterogeneity between studies (I^2^ = 0.00%, *P* = 0.83), and a random‐effects model was used for meta‐analysis. The incidence of postop headache was 0.00% [95% CI (0.0000, 0.0027)] (Fig. 2E).

##### Inadequate Decompression Rate

A total of four studies reported inadequate decompression during surgery,^11^, ^14^, ^19^, ^20^ and there was no heterogeneity among studies (I^2^ = 0.00%, *P* = 0.99). A random effects model was used for meta‐analysis. The incidence of inadequate decompression was 0.00% [95% CI (0.0000, 0.0036)] (Fig. 2F).

##### Root Injury Rate

One study reported root injury during surgery,^31^ and there was no heterogeneity among studies (I^2^ = 0.00%, *P* = 0.99). A random‐effects model was used for meta‐analysis. The incidence of root injury was 0.00% [95% CI (0.0000, 0.0022)] (Fig. 2G).

##### Infection Rate

One study reported infection during surgery,^31^ and there was no heterogeneity among studies (I^2^ = 0.00%, *P* = 1.00). A random‐effects model was used for meta‐analysis. The incidence of infection was 0.00% [95% CI (0.0000, 0.0013)] (Fig. 2H).

### Sensitivity Analysis

**Fig. 3:**
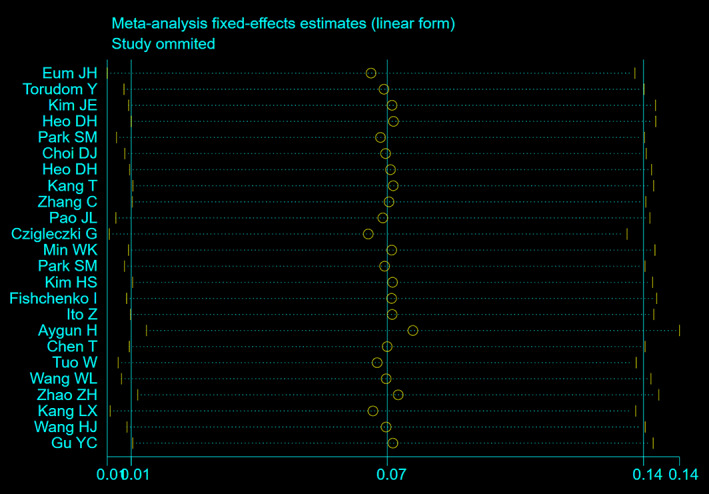
Sensitivity analysis of overall complication

Sensitivity analysis showed that none of the studies greatly interfered with the results, the sensitivity analysis results showed in Fig. 3.

### Publication Bias

**Fig. 4:**
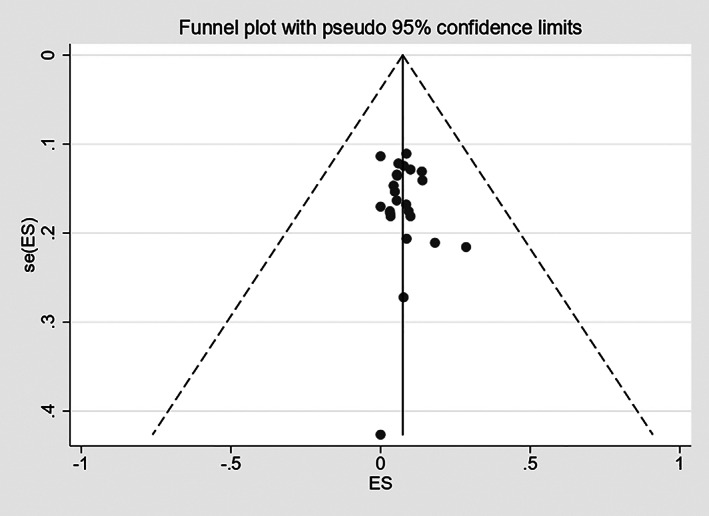
Funnel plot of publication bias for overall complication

In this study, the funnel chart was used to analyze the publication bias of total complication, and the Egger's test^15^ was used to statistically test the degree of asymmetry of the funnel chart. The results showed that there was no publication bias in the total complications (*P* = 0.454 > 0.05). The Egger's test results were 0.76 [95% CI (−0.38, 0.82)], and the funnel diagram is shown in Fig. 4.

## Discussion

This study was a single‐arm meta‐analysis of surgical complication rates for LSS treated with UBE technology. A total of 24 studies including 999 patients were included. As a minimally invasive surgical method that is gradually being promoted in clinical practice, UBE combines the advantages of traditional minimally invasive techniques and open surgery. However, due to the novelty of this technique, there may be some difficulties that hinder the promotion of the technique. Therefore, this study summarizes the surgical complications of UBE in the treatment of LSS through meta‐analysis, hoping to provide help for clinical promotion. The results showed that the total complication rate of UBE in the treatment of LSS was about was 6.27%. Complications include postop headache, transient paresthesia, dural tear, PSEH, root injury, infection, and inadequate decompression. These articles and data provide valuable experience for the follow‐up clinical UBE operation, and also analyze the causes and precautions that lead to these complications as follows.

### Dural Tear

Among them, the incidence of spinal dural tear is about 2.49%. There are four main reasons for spinal dural tear caused by UBE spine endoscopy: (i) The visual field under endoscopy is a two‐dimensional plane and is easily blurred., Beginners are easy to make mistakes;^35^ (ii) UBE does not require retraction of the anatomical structure to expose the dura mater, which is quite different from other techniques;^36^ (iii) patients with complex conditions require too long operation time and increase the risk of spinal membrane tears^20^, ^37^; (4) There is a spinal ligament that connects the dorsal side of the dura mater with the lamina and the ligamentum flavum on the surface of the central area of ​​the dura mater. This ligament is a mesh structure with thickness and shape ranging from thin strips to thick sheets It is covered and hidden by the epidural fat. During the operation, the injected saline will squeeze both sides of the dura mater, causing the area to fold. At this time, during the ligamentum flavum resection, the central area may be damaged. Spinal dura tear;^38^, ^40^ and (v) when using high‐speed drills, the peripheral fibrous bands and vascular bundles of the dura may be stretched around the drill neck, causing larger tears.^39^, ^40^, ^41^

### PSEH

PSEHs incidence is about 0.27%. The use of an infusion pump during surgery may be an unavoidable risk factor. When the outflow of saline solution is blocked, the pump continues to infuse saline to increase the pressure in the surgical field, cover up bleeding points, and cause intraoperative hemostasis. Lack of saline solution may cause PSEH.^42^ Although symptomatic PSEH is relatively rare (the incidence rate is 0.02% to 4.6%),^43^ it can lead to serious consequences such as cauda equina syndrome and even lower limb paralysis, which affects the life of patients. Therefore, early detection and early detection are important. Handling is extremely important.

### Transient Paresthesia

The incidence of transient paresthesia is about 0.14%. The main reason for transient paresthesia after surgery is that palsy and pain are both conducted by sensory nerves. The pain is transmitted by small unmyelinated fibers, and the conductive palsy is thick and thick.^10^, ^44^, ^45^ The structure of myelin fibers, unmyelinated fibers is relatively simple, and the postoperative recovery is faster, while myelin fibers need to undergo a longer and more complex repair process; in addition, pain is a more acute and uncomfortable sensation than palsy, so surgery the former is often covered up. After the postoperative pain is weakened or recovered, the palsy is exposed.^46^, ^47^, ^48^ Most patients will relieve the palsy. However, because the palsy is positively related to the illness time and the degree of stenosis, the recovery time of different patients is different.^49^

### Comparing with Previously Published Clinical Meta‐Analysis

There are three clinical studies on meta‐analysis. The article by Lin *et al*.^50^ showed that the complication rate of UBE in the treatment of LSS was 6.7%, which is higher than our study (6.27%), and the disadvantage of this article is that it only averages the results of the included articles, and is for a variety of lumbar spine diseases, not specifically for LSS, and does not analyze specific complications; Pairuchvej *et al*. and Pranata *et al*.^51^, ^52^ both concluded that 6.2%, But did not give analysis results for specific complications, and because the articles included in the above three studies are controlled studies, the number of articles is small, and the results are less convincing.

### Strengths and Limitations

So far, this is the first summary of the postoperative complications of UBE treatment of LSS, and put forward the causes for the complications. Unlike previously published clinical meta‐analysis, our latest article retrieves 16 eligible clinical studies, including controlled and single‐arm studies, and it provides more high‐level literature through evidence‐based medical analysis to obtain more reliable data from raw data. The results are designed to arouse the attention of clinicians and provide advice on surgical procedures and postoperative prevention.

This meta‐analysis has certain limitations: (i) the single‐group rate meta‐analysis has its own methodological limitations, because it is a comparison of rates, not a simple mean, so although weight indicators are added, there is no design for controlled studies, the accuracy of the results is more likely to be reduced;^9^ (ii) there are a large number of studies in South Korea included in this study, and the extrapolation of the results has certain limitations; and (iii) some studies only conduct descriptive analysis and cannot extract accurate clinical data. In short, there are certain postoperative complications in the treatment of LSS with UBE. Limited by the number and quality of the studies included, the above conclusions still need to be confirmed by more studies.

### Conclusions

Our systematic review of the meta‐analysis concluded that the UBE technique has a lower postoperative complication rate in general, and is safer than other minimally invasive techniques that have been reported. It is worth noting that, the incidence of spinal dural tears is higher than other complications (2.49%) and requires more detailed surgical operations. At the same time, because epidural PSEH may cause serious complications, timely follow‐up is required after an imaging review after surgery.

## Author Contributions

Wang Bin, He Peng, and Wu Zhengfang designed the systematic review. Wang Bin and He Peng drafted the protocol, and Liu Xiaowei and Xu Bin revised the manuscript. Wang Bin and He Peng will independently screen the potential studies, extract data, assess the risk of bias, and finish data synthesis. Wang Bin and He Peng will arbitrate any disagreements during the review. All authors approved the publication of the manuscript.

## References

[1] Jenis LG , An HS . Spine update: lumbar foraminal stenosis. Spine. 2000;25(3):389–94. 10703115 10.1097/00007632-200002010-00022

[2] Ahn Y . Current techniques of endoscopic decompression in spine surgery. Ann Transl Med. 2019;7(Suppl 5):S169. 31624735 10.21037/atm.2019.07.98 PMC6778275

[3] Ahn JS , Lee HJ , Park EJ , et al. Multifidus muscle changes after biportal endoscopic spinal surgery: magnetic resonance imaging evaluation. World Neurosurg. 2019;130:e525–34. 31254694 10.1016/j.wneu.2019.06.148

[4] Choi CM . Biportal endoscopic spine surgery (BESS): considering merits and pitfalls. J Spine Surg. 2020;6(2):457–65. 32656383 10.21037/jss.2019.09.29 PMC7340825

[5] Kambin P , Sampson S . Posterolateral percutaneous suction‐excision of herniated lumbar intervertebral discs. Report of interim results. Clin Orthop Relat Res. 1986;207:37–43. 3720102

[6] Choi CM , Chung JT , Lee SJ , et al. How I do it? Biportal endoscopic spinal surgery (BESS) for treatment of lumbar spinal stenosis. Acta Neurochir. 2016;158(3):459–63. 26782827 10.1007/s00701-015-2670-7 PMC4752582

[7] Slim K , Nini E , Forestier D , et al. Methodological index for non‐randomized studies (minors): development and validation of a new instrument. ANZ J Surg. 2003;73(9):712–6. 12956787 10.1046/j.1445-2197.2003.02748.x

[8] Freeman MF , Tukey JW . Transformations related to the angular and the square root. Ann Math Stat. 1950;21(4):607–11.

[9] Schwarzer G , Chemaitelly H , Abu‐Raddad LJ , et al. Seriously misleading results using inverse of Freeman‐Tukey double arcsine transformation in meta‐analysis of single proportions. Res Synth Methods. 2019;10(3):476–83. 30945438 10.1002/jrsm.1348 PMC6767151

[10] Eum JH , Heo DH , Son SK , et al. Percutaneous biportal endoscopic decompression for lumbar spinal stenosis: a technical note and preliminary clinical results. J Neurosurg Spine. 2016;24(4):602–7. 26722954 10.3171/2015.7.SPINE15304

[11] Torudom Y , Dilokhuttakarn T . Two portal percutaneous endoscopic decompression for lumbar spinal stenosis: preliminary study. Asian Spine J. 2016;10(2):335–42. 27114776 10.4184/asj.2016.10.2.335 PMC4843072

[12] Kim JE , Choi DJ . Clinical and radiological outcomes of unilateral biportal endoscopic decompression by 30 degrees arthroscopy in lumbar spinal stenosis: minimum 2‐year follow‐up. Clin Orthop Surg. 2018;10(3):328–36. 30174809 10.4055/cios.2018.10.3.328 PMC6107815

[13] Heo DH , Javier QO , Park CK . Can percutaneous biportal endoscopic surgery achieve enough canal decompression for degenerative lumbar stenosis? Prospective case‐control study. World Neurosurg. 2018;120:e684–9. 30165228 10.1016/j.wneu.2018.08.144

[14] Park SM , Kim HJ , Kim GU , et al. Learning curve for lumbar decompressive laminectomy in biportal endoscopic spinal surgery using the cumulative summation test for learning curve. World Neurosurg. 2019;122:e1007–13. 30404053 10.1016/j.wneu.2018.10.197

[15] Choi DJ , Kim JE . Efficacy of biportal endoscopic spine surgery for lumbar spinal stenosis. Clin Orthop Surg. 2019;11(1):82–8. 30838111 10.4055/cios.2019.11.1.82 PMC6389528

[16] Heo DH , Lee DC , Park CK . Comparative analysis of three types of minimally invasive decompressive surgery for lumbar central stenosis: biportal endoscopy, uniportal endoscopy, and microsurgery. Neurosurg Focus. 2019;46(5):E9. 10.3171/2019.2.FOCUS197 31042664

[17] Kang T , Park SY , Kang CH , et al. Is biportal technique/endoscopic spinal surgery satisfactory for lumbar spinal stenosis patients? A prospective randomized comparative study. Medicine. 2019;98(18):e15451. 31045817 10.1097/MD.0000000000015451 PMC6504265

[18] Zhang C , Jian FZ , Chen Z . Preliminary clinical research of biportal endoscopic surgery for lumbar spinal canal stenosis. Chin J Minim Invasive Neurosurg. 2019;24(06):260–3.

[19] Pao JL , Lin SM , Chen WC , et al. Unilateral biportal endoscopic decompression for degenerative lumbar canal stenosis. J Spine Surg. 2020;6(2):438–46. 32656381 10.21037/jss.2020.03.08 PMC7340817

[20] Czigleczki G , Nagy Z , Padanyi C , et al. Biportal endoscopic technique in the treatment of spinal stenosis: early clinical experiences and results. Neurol Res. 2020;42(12):1085–8. 32892719 10.1080/01616412.2020.1803603

[21] Min WK , Kim JE , Choi DJ , et al. Clinical and radiological outcomes between biportal endoscopic decompression and microscopic decompression in lumbar spinal stenosis. J Orthop Sci. 2020;25(3):371–8. 31255456 10.1016/j.jos.2019.05.022

[22] Park SM , Park J , Jang HS , et al. Biportal endoscopic versus microscopic lumbar decompressive laminectomy in patients with spinal stenosis: a randomized controlled trial. Spine J. 2020;20(2):156–65. 31542473 10.1016/j.spinee.2019.09.015

[23] Kim HS , Choi SH , Shim DM , et al. Advantages of new endoscopic unilateral laminectomy for bilateral decompression (ULBD) over conventional microscopic ULB. Clin Orthop Surg. 2020;12(3):330–6. 32904063 10.4055/cios19136 PMC7449863

[24] Fishchenko I , Kravchuk L , Saponenko A , et al. Experience of biportal endoscopic decompression in lumbar spinal stenosis. Georgian Med News. 2020;303:21–7. 32841175

[25] Ito Z , Shibayama M , Nakamura S , et al. Clinical comparison of unilateral biportal endoscopic laminectomy versus microendoscopic laminectomy for single‐level laminectomy: A single‐center, retrospective analysis. World Neurosurg. 2021;148:e581–8. 33476779 10.1016/j.wneu.2021.01.031

[26] Aygun H , Abdulshafi K . Unilateral biportal endoscopy versus tubular microendoscopy in management of single level degenerative lumbar canal stenosis: a prospective study. Clin Spine Surg. 2021;34(6):E323–8. 33470660 10.1097/BSD.0000000000001122 PMC8225231

[27] Chen T , Wang QN , Zhang C , et al. Observation on curative effect of spinal canal decompression with unilateral biportal endoscopic technology in treatment of lumbar spinal stenosis. Chin J Bone Jt Injury. 2021;36(09):905–8.

[28] Tuo W , Zhou L , Liu DS , et al. Preliminary study of unilateral biportal endoscopic treatment of lumbar spinal stenosis. Chin J Min Inv Surg. 2021;21(01):56–60.

[29] Wang WL , Liu Z , Wu SJ , et al. Preliminary clinical outcomes of unilateral biportal endoscopy for decompressing lumbar spinal stenosis. Chin J Spine Spinal Cord. 2021;31(10):911–8.

[30] Zhao ZH , Li XC , Li JJ , et al. Unilateral biportal endoscopic for lumbar spinal stenosis. BMU J. 2021;44(05):341–5.

[31] Kang LX , Yang SM , Zhang P , et al. Comparison of clinical efficacy of single channel, unilateral double channel and bilateral three channel spinal endoscopy in the treatment of lumbar spinal stenosis. J North Sichuan Med Coll. 2021;36(10):1323–8.

[32] Wang HJ , Wu ZP . Comparison of single channel‐spinal endoscopy (Delta) and unliateral biportal endoscopic technique for the treatment of senile lumbar spinal stenosis. J Xi'an Jiaotong Univ. 2021;42(06):791–801.

[33] Gu YC , Li Y , Xie W , et al. Clinical analysis of unilateral biportal endoscopy for treatment of lumbar spinal stenosis. Int J Orthop. 2021;42(05):323–8.

[34] Egger M , Davey Smith G , Schneider M , et al. Bias in meta‐analysis detected by a simple, graphical test. BMJ. 1997;315(7109):629–34. 9310563 10.1136/bmj.315.7109.629 PMC2127453

[35] Choi DJ , Choi CM , Jung JT , et al. Learning curve associated with complications in biportal endoscopic spinal surgery: challenges and strategies. Asian Spine J. 2016;10(4):624–9. 27559440 10.4184/asj.2016.10.4.624 PMC4995243

[36] Choi DJ , Kim JE , Jung JT , et al. Biportal endoscopic spine surgery for various foraminal lesions at the lumbosacral lesion. Asian Spine J. 2018;12(3):569–73. 29879787 10.4184/asj.2018.12.3.569 PMC6002165

[37] Heo DH , Ha JS , Lee DC , et al. Repair of incidental durotomy using sutureless nonpenetrating clips via biportal endoscopic surgery. Global Spine J. 2022;12(3):452–7. 33148035 10.1177/2192568220956606 PMC9121153

[38] Heo DH , Sharma S , Park CK . Endoscopic treatment of extraforaminal entrapment of L5 nerve root (far out syndrome) by unilateral biportal endoscopic approach: technical report and preliminary clinical results. Neurospine. 2019;16(1):130–7. 30943715 10.14245/ns.1938026.013 PMC6449829

[39] Hong YH , Kim SK , Suh DW , et al. Novel instruments for percutaneous biportal endoscopic spine surgery for full decompression and dural management: a comparative analysis. Brain Sci. 2020;10(8):516. 32759697 10.3390/brainsci10080516 PMC7463780

[40] Kim W , Kim SK , Kang SS , et al. Pooled analysis of unsuccessful percutaneous biportal endoscopic surgery outcomes from a multi‐institutional retrospective cohort of 797 cases. Acta Neurochir. 2020;162(2):279–87. 31820196 10.1007/s00701-019-04162-2

[41] Kang MS , Park HJ , Hwang JH , et al. Safety evaluation of biportal endoscopic lumbar discectomy: assessment of cervical epidural pressure during surgery. Spine. 2020;45(20):E1349–56. 32969993 10.1097/BRS.0000000000003585

[42] Kang MS , Hwang JH , Choi DJ , et al. Clinical outcome of biportal endoscopic revisional lumbar discectomy for recurrent lumbar disc herniation. J Orthop Surg Res. 2020;15(1):557. 33228753 10.1186/s13018-020-02087-6 PMC7685633

[43] Awad JN , Kebaish KM , Donigan J , et al. Analysis of the risk factors for the development of post‐operative spinal epidural haematoma. Bone Jt Surg Br. 2005;87(9):1248–52. 10.1302/0301-620X.87B9.16518 16129751

[44] Kang T , Park SY , Lee SH , et al. Spinal epidural abscess successfully treated with biportal endoscopic spinal surgery. Medicine. 2019;98(50):e18231. 31852084 10.1097/MD.0000000000018231 PMC6922448

[45] Kim HS , Wu PH , Jang IT . Lumbar endoscopic unilateral laminotomy for bilateral decompression outside‐in approach: a proctorship guideline with 12 steps of effectiveness and safety. Neurospine. 2020;17(Suppl 1):S99–S109. 32746523 10.14245/ns.2040078.039 PMC7410378

[46] Kim JE , Choi DJ , Park EJ . Evaluation of postoperative spinal epidural hematoma after biportal endoscopic spine surgery for single‐level lumbar spinal stenosis: clinical and magnetic resonance imaging study. World Neurosurg. 2019;126:e786–92. 30878758 10.1016/j.wneu.2019.02.150

[47] Kim JE , Yoo HS , Choi DJ , et al. Effectiveness of gelatin‐thrombin matrix sealants (Floseal(R)) on postoperative spinal epidural hematoma during single‐level lumbar decompression using biportal endoscopic spine surgery: clinical and magnetic resonance image study. Biomed Res Int. 2020;4801641. 32695815 10.1155/2020/4801641 PMC7368184

[48] Kim KR , Park JY . The technical feasibility of unilateral biportal endoscopic decompression for the unpredicted complication following minimally invasive transforaminal lumbar interbody fusion: case report. Neurospine. 2020;17(Suppl 1):S154–9. 32746529 10.14245/ns.2040174.087 PMC7410383

[49] Kim N , Jung SB . Percutaneous unilateral biportal endoscopic spine surgery using a 30‐degree arthroscope in patients with severe lumbar spinal stenosis: a technical note. Clin Spine Surg. 2019;32(8):324–9. 31464695 10.1097/BSD.0000000000000876 PMC6791497

[50] Lin GX , Huang P , Kotheeranurak V , et al. A Systematic Review of Unilateral Biportal Endoscopic Spinal Surgery: Preliminary Clinical Results and Complications. World Neurosurg. 2019;125:425–32. 30797907 10.1016/j.wneu.2019.02.038

[51] Pairuchvej S , Muljadi JA , Ho JC , et al. Full‐endoscopic (bi‐portal or uni‐portal) versus microscopic lumbar decompression laminectomy in patients with spinal stenosis: systematic review and meta‐analysis. Eur J Orthop Surg Traumatol. 2020;30(4):595–611. 31863273 10.1007/s00590-019-02604-2

[52] Pranata R , Lim MA , Vania R , et al. Biportal endoscopic spinal surgery versus microscopic decompression for lumbar spinal stenosis: a systematic review and meta‐analysis. World Neurosurg. 2020;138:e450–8. 32147545 10.1016/j.wneu.2020.02.151

